# Focused cardiopulmonary ultrasound for assessment of dyspnea in a resource-limited setting

**DOI:** 10.1186/s13089-016-0043-y

**Published:** 2016-06-03

**Authors:** Sachita P. Shah, Sachin P. Shah, Reginald Fils-Aime, Walkens Desir, Joanel Joasil, David M. Venesy, Krithika Meera Muruganandan

**Affiliations:** Division of Emergency Medicine, Harborview Medical Center, University of Washington School of Medicine, Box 359702, 325 9th Ave, Seattle, WA 98104 USA; Division of Cardiology, Lahey Hospital and Medical Center, Burlington, MA USA; Hopital Bon Sauveur, Zanmi Lasante/Partners In Health, Central Plateau, Cange, Haiti; Department of Emergency Medicine, Boston Medical Center, Boston, MA USA

**Keywords:** Heart failure, Ultrasound, Point-of-care, Resource-limited

## Abstract

**Background:**

The diagnosis and management of acutely dyspneic patients in resource-limited developing world settings poses a particular challenge. Focused cardiopulmonary ultrasound (CPUS) may assist in the emergency diagnosis and management of patients with acute dyspnea by identifying left ventricular systolic dysfunction, pericardial effusion, interstitial pulmonary edema, and pleural effusion. We sought to assess the accuracy of emergency providers performing CPUS after a training intervention in a limited-resource setting; a secondary objective was to assess the ability of CPUS to affect change of clinician diagnostic assessment and acute management in patients presenting with undifferentiated dyspnea.

**Methods and results:**

After a training intervention for Haitian emergency providers, patients with dyspnea presenting urgently to a regional referral center in Haiti underwent a rapid CPUS examination by the treating physician. One hundred seventeen patients (median age of 36 years, 56 % female) were prospectively evaluated with a standardized CPUS exam. Blinded expert review of ultrasound images was performed by two board certified cardiologists and one ultrasound fellowship trained emergency physician. Inter-observer agreement was determined using an agreement coefficient (kappa). Sensitivity and Specificity with confidence intervals were calculated. Pre-test and post-test clinician impressions and management plans were compared to assess for a change. We enrolled 117 patients with undifferentiated dyspnea. Upon expert image review, prevalence of left ventricular systolic dysfunction was 40.2 %, and in those with systolic dysfunction, the average EF was 14 % (±9 %). The parasternal long axis (PLAX) single view was predictive of an overall abnormal echo with PPV of abnormal PLAX 95 % and NPV 93 % of normal PLAX. Weighted kappa for pericardial effusion between the Haitian physicians and two cardiology reviewers was 0.81 (95 % CI 0.75–0.87, *p* value <0.001) and for ejection fraction was 0.98 (95 % CI 0.98–0.99, *p* value <0.001). For lung ultrasound, a kappa statistic assessing agreement between the Haitian physician and the EP for pleural effusion was 0.73, and for interstitial syndrome was 0.49. Detailed test characteristics are detailed in Table 3. Overall, there was a change in treating clinician impression in 15.4 % (95 % CI 9–22 %) and change in management in 19.6 % (95 % CI 12–27 %) of patients following CPUS. A significant structural heart disease was common: 48 % of patients were noted to have abnormal right ventricular systolic function, 36 % had at least moderate mitral regurgitation, and 7.7 % had a moderate to large pericardial effusion.

**Conclusions:**

A focused training intervention in CPUS was sufficient for providers in a limited-resource setting to accurately identify left ventricular systolic dysfunction, pericardial effusion, evidence of interstitial syndrome, and pleural effusions in dyspneic patients. Clinicians were able to integrate CPUS into their clinical impressions and management plans and reported a high level of confidence in their ultrasound findings.

## Background

Clinicians’ world-wide rise to the diagnostic challenge presented by patients with undifferentiated dyspnea, relying on both clinical acumen and diagnostic testing to determine diagnosis. The differential diagnosis of acute dyspnea in limited-resource international settings is broad, including diagnoses in which point-of-care-ultrasound findings can influence overall assessment and acute management, such as acute decompensated heart failure (ADHF), pericardial effusion with tamponade, and large pleural effusions causing respiratory distress. The burden of heart failure in particular in the developing world is likely significant, but has not been sufficiently characterized, and resource limitations in these settings impose an obstacle to early disease recognition and appropriate therapy. Cardiopulmonary ultrasound (CPUS) is a portable and reusable resource that may be useful in establishing a diagnosis in patients presenting with acute dyspnea.

Improvements in the portability and cost of ultrasound equipment have facilitated an increase in its utilization for the rapid identification of pathology in several clinical and non-clinical settings [1–8]. CPUS has been used effectively in emergency and intensive care settings in the evaluation of the dyspneic patient [9–12]. There is sufficient evidence to support the adequacy of brief focused instruction of physicians in focused cardiac ultrasound and basic lung ultrasound [7, 10, 13], however, ours is the first study to demonstrate this type of training intervention in a resource-limited setting. In addition, no prior study has addressed whether focused ultrasound findings at the point-of-care will actually influence clinicians to change their diagnosis or management plans of acutely dyspneic patients.

**Fig. 1:**
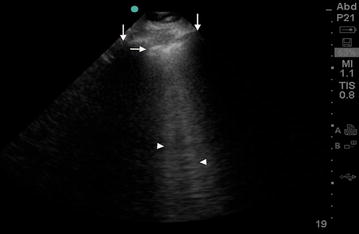
Example of B-lines. The pleural line (*horizontal arrow*) is identified as an echogenic line noted about 0.5-cm deep and perpendicular to the rib shadowing (*vertical arrows*). The comet-tail or ring-down artifacts (*arrow heads*) begin at the pleural line and extend to the edge of the image

Ultrasound is central to the evaluation of common causes of dyspnea. While heart failure is a syndrome of clinical signs and symptoms, echocardiography is helpful in the diagnosis and further characterization of patients with suspected ADHF. Lung ultrasound is emerging as an innovative and simple tool to identify extravascular lung water (alveolar interstitial edema and pleural effusion), pneumothorax, and alveolar consolidation [14–17]. B-lines are ultrasound comet-tailed artifacts which relate to subpleural interstitial edema (Fig. 1) [11, 17–19]. Excessive B-lines have been found to correlate with a diagnosis of ADHF, chest roentgenogram findings of pulmonary edema, brain natriuretic peptide (BNP) levels, and pulmonary arterial wedge pressure [12, 20–22]. These artifacts are easily identified with lung ultrasound of the anterior and lateral chest, are quantifiable, and rapidly resolve with decongestion [23, 24].

We have developed a rapid CPUS protocol to assist physicians practicing in remote, austere environments in evaluating patients presenting urgently with dyspnea. We conducted a prospective study in a single large hospital in Haiti’s remote Central Plateau to characterize ability of providers to accurately perform focused cardiac and lung ultrasound after a novel training intervention, and to assess whether that information was integrated into the treating clinician’s overall impression, such that a change in impression and/or management plans was initiated.

## Methods

### Study population

This study is a prospective cohort analysis of patients presenting with dyspnea for the urgent medical evaluation at the Hospital Bon Sauveur, a 104 bed regional referral center, in Haiti’s remote Central Plateau. This study was approved by the internal review board of Zamni Lasante (Partners In Health) and all subjects provided informed consent. All adult and pediatric patients presenting to the emergency department or urgently to outpatient clinics with acute dyspnea were eligible for this study. Patients were enrolled from February to August 2012 within 24 h of presentation. Patients were excluded if they had an echocardiogram in the preceding year.

### Study protocol

Patients presenting with undifferentiated dyspnea were evaluated by the treating physician with a detailed history, physical examination, and limited laboratory, and radiographic testing. A preliminary diagnosis and management plan were recorded by the treating clinician. The treating physician then performed a CPUS examination (Table 1) and recorded a post-test clinical impression and management plan. In addition, the level of confidence of the performing physician was self-reported using a five-point Likert scale. Admitted patients were followed until discharge from the hospital noting any procedures, complications, and the final discharge diagnosis.

**Table 1:** Cardiopulmonary ultrasound protocol

*Echocardiogram*	
Parasternal long axis with color Doppler	Estimate LVEF Pericardial effusion Mitral valve disease
Parasternal short axis (mid-ventricle)	
Apical 4 chamber with color Doppler	
Subcostal	
*Pulmonary ultrasound*	
Anterior and lateral chest ultrasound, rib interspaces 2–4 on left and 2–5 on right for upper zones, and rib spaces below 5 for lower zones bilaterally	Extravascular lung water (B-lines) Pleural effusion Pneumothorax (lack of lung sliding)

*LVEF* left ventricular ejection fraction

### Ultrasound training and CPUS protocol

Focused cardiac ultrasound and lung ultrasound were performed by seven Haitian physicians (internal medicine residency trained and social service residents) who underwent a 3-week ultrasound training program in point-of-care-ultrasound. The ultrasound training program entailed 10 h of structured didactic training and 20 h of supervised practice. All examinations were performed using a Sonosite Micromaxx with a phased array (P17) transducer (Sonosite Inc, Bothell, WA). The focused cardiac ultrasound included 6-second acquisitions of each the following: parasternal long axis view, parasternal short axis view at the mid-ventricular level, apical four chamber view with and without color Doppler, and subcostal four chamber view (Table 1). The treating physician was required to assess left ventricular ejection fraction (LVEF) by visual estimation, presence and size of pericardial effusion, and presence and characterization of mitral valve disease, including regurgitation using color flow and stenosis. Mitral regurgitation was defined as color flow back through the closed mitral valve during systole. The presence of mitral stenosis is difficult to assess in the absence of continuous wave Doppler, and in our study, was estimated based on visual assessment of the mitral valve in both parasternal long axis (PLAX) and parasternal short axis (PSS) view for classic findings of mitral stenosis, including valve leaflet thickening, hockey stick deformity (PLAX), fish mouth appearance (PSS), and reduced leaflet movement of both the two dimensional images, as well as prominent antegrade flow acceleration through the mitral valve during diastole using a standard Nyquist limit. The practitioners were not trained in calculating/estimating valve area or gradient based on the spectral Doppler analysis; therefore, the presence or absence of mitral stenosis was based on the above parameters and is limited to detection of severe mitral stenosis.

Lung ultrasound was performed using the same transducer. Ultrasound of the anterior and lateral chest bilaterally in the supine or recumbent position perpendicular to the ribs was performed to assess for interstitial syndrome as characterized by the presence of increased numbers of B-lines, defined as moderately hyperechoic, vertical, well-defined, dynamic lines originating from the pleural line, and extending deep to the bottom of the screen [18, 25]. Interstitial syndrome was considered present if ≥3 B-lines were noted in two or more rib spaces anteriorly or laterally. At least two rib spaces were scanned in each zone of the chest, and zones were upper/superior anterior, lower/inferior anterior, upper/superior lateral, and lower/inferior lateral. Ultrasound of the mid-axillary line at the level of the costophrenic recesses bilaterally was performed to evaluate for pleural effusion.

### Outcome measures and data interpretation

To assess our primary outcome of determining accuracy of trained health providers in Haiti using CPUS in the evaluation of dyspnea, three independent reviewers assessed the recorded ultrasound images produced by the trained subject physicians. For the focused cardiac ultrasound video clips, blinded review was conducted independently by two board certified cardiologists with experience teaching ultrasound in the developing world and specialty training in echocardiography and heart failure (HF). For the lung ultrasound recording, blinded review was conducted by an emergency ultrasound fellowship trained EP. Reviewers were blinded to the patient characteristics as well as the study physician’s interpretation of the ultrasound exams.

For the quality assessment, blinded reviewers rated the ultrasound image quality using a 1–5 scale published in the American College of Emergency Physicians Emergency Ultrasound standard reporting guidelines [26]. This scale uses ratings of 1 and 2 for images with insufficient data for diagnosis, and 3–5 for images that are sufficient for diagnosis with improving image quality throughout the scale.

For accuracy assessment and establishment of test characteristics, cardiologist review of echocardiography is essential for the formal diagnosis of HF; therefore, this was considered the criterion standard for the assessment of left ventricular systolic dysfunction [27, 28]. Similarly, because several studies have established high sensitivity of emergency physician interpretation of bedside ultrasound for the diagnosis of pleural effusion, pneumothorax, and pathologic B-lines as referenced above, and due to the limited resources available at the study site (limited laboratory tests, limited plain film radiography, lack of formal ultrasound and CT), blinded review of the lung ultrasound videos was used as the criterion standard for determining presence or absence of pleural effusion, and B-lines [11, 16, 29].

Considering these criterion standards, kappas for inter-observer agreement between both expert reviewers and the point-of-care study physician interpretation, as well as and performance characteristics (sensitivity SN, and specificity SP) were calculated for each ultrasound exam type, and confidence levels were evaluated. The same criteria for the diagnosis of cardiac and pulmonary sonographic abnormalities were used by the Haitian clinicians and the expert reviewers.

For the evaluation of the secondary outcome of CPUS in influencing clinicians change in management and clinical impression, pre-test and post-test diagnoses by the treating clinicians were compared, and frequency of change in the treating clinicians’ impressions and management plans was calculated.

### Statistical analysis

We calculated our sample size based on our primary outcome of assessing accuracy of clinicians to use CPUS at the point-of-care to accurately assess the common causes of dyspnea. Though there is a little available data on the prevalence of the top causes of dyspnea in tropical, resource-poor medical settings, we focused on powering our study to demonstrate ability of clinicians to detect left ventricular systolic dysfunction. We designed our study to include sufficient patients, such that the prevalence of LV systolic dysfunction would be not uncommon, and to demonstrate that the sensitivity to diagnose ADHF exceeded 70 %, which we defined as the minimum clinically acceptable sensitivity. Under the hypothesis that subject physicians would have 90 % sensitivity, a sample size of 40 patients with disease would have 90 % power with the exact binomial test of proportions at alpha = 0.025 (one-sided). Given a prevalence of HF in prior studies at 21–58 %, we estimated that a total of 110 dyspneic patients would be necessary to yield 40 patients with HF as a cause for their dyspnea [30–34].

Categorical data were analyzed using Fisher’s exact test and continuous data using a student *t*-test. A weighted kappa measurement was calculated for assessing agreement between the physician performing the CPUS and the expert reviewer.

## Results

A total of 117 patients were enrolled in the study over a 7-month period. Twenty nine (24.8 %) of the patients were children under the age of 18, the median age of the overall study cohort was 36 years (interquartile range 17–58 years). Of the pediatric patients, the average age was 3.4 years (range 2 months–17 years), and 22 of our 29 children were under 5 years old. Known comorbid conditions were not common and are detailed in Table 2. Fifty-six percent of patients were female. Most of the patients (81 %) were admitted to the hospital, with a median length of hospitalization of 8 days. Of the 95 patients admitted to the hospital, 17 (17.9 %) died during their hospitalization. There were no deaths in the hospitalized pediatric patients. The final diagnoses as determined by the treating clinicians as the charted discharge diagnosis (based on clinical presentation, laboratory studies, and chest film findings, response to treatment and ultrasound findings) of the enrolled patients are illustrated in Table 2. The most common final discharge diagnosis in adults was HF (*N* = 40/88), and for pediatric patients, pneumonia was the most frequent discharge diagnosis (*N* = 16/29).

**Table 2:** Demographic data, clinical findings, and outcomes

	Total patients *N* = 117	Pediatric (<18 years) *N* = 29	Adult (>18 years) *N* = 88
Female	65 (56 %)	11 (38 %)	54 (61 %)
*Comorbid conditions*			
Tobacco	9 (8 %)	0	9 (10 %)
Post-partum	8 (7 %)	0	8 (9 %)
Tuberculosis	5 (4 %)	0	5 (6 %)
HIV	5 (4 %)	1	4 (5 %)
Diabetes mellitus	2 (2 %)	0	2 (2 %)
*Clinical findings*			
Tachypnea (*n* = 88)	88 (100 %)	NA*	88 (100 %)
Tachycardia (*n* = 86)	57 (66 %)	NA*	57 (66 %)
Abnormal lung exam	83 (71 %)	27 (93 %)	56 (63 %)
Peripheral edema	44 (38 %)	0	44 (50 %)
*Outcomes*			
Hospitalized	95 (81 %)	28 (97 %)	67 (76 %)
Median/IQR length of hospital stay (days)	6/6 (n = 92)	6/4 (*n* = 26)	6/8 (*n* = 66)
Death during hospitalization	17 (15 %)	0	17 (19 %)

Data are reported as number of patients (percentage)

Post-partum is defined as delivery in the prior 6 months. Abnormal vital signs (tachypnea and tachycardia) are reported for adults only. Tachypnea is defined as respiratory rate >20 breaths per minute. Tachycardia is defined as heart rate >100 beats per minute. Abnormal lung exam was defined as decreased breath sounds, wheezes, crackles, and/or rhonchi, though was left to the interpretation of the treating clinician at the time of the exam. Pediatric heart rate and respiratory rate were recorded however given variability of vital signs with change in age, we did not define tachycardia/tachypnea for children

All percentages have been rounded to the nearest whole percent

*HIV* human immunodeficiency virus, *IQR* interquartile range, *NA* not applicable

For assessment of the primary outcome of accuracy of CPUS in a limited-resource setting, we present data on quality of images produced, and agreement compared to the criterion standard of blinded review by an expert. Of the 117 patients enrolled, all had images which were available for blinded review. One hundred eight (92.3 %) of patients had echocardiographic images considered to be of sufficient quality and 107 (91.4 %) of patients had pulmonary ultrasound images that were of sufficient quality. The average level of confidence of the performing physician (on a 5 point Likert scale, 1 = not confident and 5 = very confident) was high at 4.2.

Compared to blinded expert reviewer interpretation of ultrasound images, the treating physicians had an excellent sensitivity and specificity in detecting left ventricular (LV) systolic dysfunction (93.6 and 100 %, respectively) (Table 3), and agreement in assessing the severity of LV dysfunction was excellent (kappa = 0.98, *p* = <0.001). The agreement in the size of pericardial effusion (none, small, moderate, large) was excellent (kappa = 0.81, *p* < 0.001). The agreement in type of mitral valve disease (normal, regurgitation, stenosis, and mixed-valve disease) was good (kappa 0.7, *p* < 0.001).

**Table 3:** Cardiopulmonary ultrasound findings: prevalence and test characteristics

CPUS findings	Prevalence %	Sensitivity %	Specificity %	Kappa
Pericardial effusion	7.7 (3.8, 14.5)	88.9 (50.7, 99.4)	99.1 (94.2, 100)	0.81*
LVEF <50 %	40.2 (31.3, 49.7)	93.6 (81.4, 98.3)	100 (93.5, 100)	0.98*
MV disease	53.8 (43.2, 64.1)	86.0 (72.6, 93.7)	86.0 (71.4, 94.2)	0.696*
Pleural effusion	25.7 (20.2, 31.9)	83.1 (70.6, 91.1)	100 (97.3, 100)	0.73*
Interstitial synd.	36.3 (30.1, 43.0)	92.7 (84.2, 97.0)	97.9 (93.6, 99.5)	0.49*

Numbers are reported as a percentage (confidence interval)

*CPUS* cardiopulmonary ultrasound, *LVEF* left ventricular ejection fraction, *MV* mitral valve, *Interstitial synd* Interstitial Syndrome)

* *p* < 0.001

For assessment of the secondary objective of assessing integration of the new CPUS protocol into clinical impression and management decisions, we analyzed pre- and post-CPUS physician impression for comparison. An initial clinical impression of ADHF based on initial history and exam by the treating clinician was made in 49/117 (41.9 %) patients. The treating clinician’s impression was changed based on the results of CPUS in 11 (22.4 %) of these patients. The remaining 68 (58.2 %) patients were clinically diagnosed with a non-heart failure cause of dyspnea. This initial assessment was changed to heart failure in 7 (10.3 %) following CPUS. Overall, the treating clinicians used their CPUS results to impact change in their clinical impression in 18 (15.4, 95 % CI 9–22 %) patients.

Change in clinical impression of the treating clinician in our pediatric population was lower as expected given the high prevalence of respiratory infections as a cause of dyspnea, and lower overall presence of HF in children world-wide. Pre-CPUS none of the 29 pediatric patients were diagnosed with ADHF as a cause of dyspnea, and after CPUS, the treating clinician’s impression was changed (changed, broadened or narrowed) in 4/29 patients (13.7 %). Two children from this cohort were diagnosed with severe LV dysfunction on CPUS leading to a change in management in one patient (3.4 %).

Amongst our total study population, CPUS resulted in a change in the management strategy in 23 (19.6, 95 % CI 12–22 %) cases. One procedure (thoracentesis) was cancelled based on the ultrasound results, in the remaining 22 cases, there was a change in medical therapy. An additional 12 procedures (e.g. thoracentesis or paracentesis) were performed with the use of ultrasound guidance.

These findings suggest that not only were study clinicians able to accurately perform focused cardiac and lung ultrasound to produce high quality images, with a high level of confidence, but also that they did use this information to guide their clinical impression and management of patients at the point-of-care.

### Echocardiography

For additional descriptive statistics in our patient population, we present detailed analysis of the focused cardiac ultrasound results. Of the 117 patients, 47 (40.2 %) had evidence of LV systolic dysfunction (LVEF <50 %) on CPUS. This cohort was comprised 45 adults and 2 pediatric patients found to have LV systolic dysfunction. In the subset of patients with LV systolic dysfunction, the mean EF was 14 ± 9 %, and both pediatric patients in this cohort had an EF of 10 %. Abnormal right ventricular (RV) function was noted in 56 (47.9 %) patients. Of those with RV dysfunction, the RV dysfunction was isolated (without LV dysfunction or mitral valve disease) in 12 (21.4 %) patients and was severe in 27 (48.2 %).

Mitral regurgitation could be assessed in 93 of the 117 patients, and of this 58 (62.4 %) had at least mild mitral regurgitation, of those, 42 (72.4 %) had moderate-to-severe mitral regurgitation. In 54 (93.1 % of those of with mitral regurgitation) patients, the etiology of regurgitation was considered to be functional, only four (6.9 %) of the 58 patients with any mitral regurgitation had a rheumatic deformity of the valve. A moderate or large pericardial effusion was noted in 9 (7.7 %) patients.

A single parasternal long axis view without color Doppler was reviewed for each patient in isolation and then compared to the all echocardiogram images. Patients with a normal appearing parasternal long axis image were very unlikely to have significant structural heart disease (defined as LV or RV systolic dysfunction, moderate-to-large pericardial effusion, or more than mild mitral valve disease) detected on the remaining images. The negative predictive value of normal parasternal long axis was 93 %.

### Lung ultrasound

Lung ultrasound revealed pleural effusions in 30 (25.7 %) patients, all adults. B-lines as defined as ≥3 B-lines found in two or more rib spaces anteriorly or laterally were noted in 42 (35.9 %) patients, including 7 children and 35 adults. Of note, B-lines were diffuse in all lung zones in two of the pediatric patients, those found to have ADHF, and focal in the remaining five pediatric patients, all subsequently diagnosed with pneumonia as their final diagnosis. Using expert interpretation of ultrasound as the reference standard, the sensitivity and specificity of the performing physician’s interpretation for pleural effusion were 83.1 and 100 %, and for B-lines was 92.7 and 97.9 %, respectively.

## Discussion

Our study suggests that providers in an international, limited-resource setting can perform CPUS after a relatively brief training with a high degree of accuracy to detect sonographically evident potential causes of dyspnea, such as left ventricular systolic dysfunction and pericardial effusion. To our knowledge, this is the first study of the impact of a novel curriculum for a focused point-of-care-ultrasound protocol for dyspnea to be conducted in a limited-resource setting.

Using a similar curriculum to those used in the US emergency medicine residency programs, we developed a training intervention for focused cardiac and lung ultrasound and included not only training for common causes of dyspnea world-wide, such as HF and pericardial effusion, but also included specialized training for rheumatic valvular disease [35]. Our study training period was relatively brief, 3 weeks, but included 20 h of hands-on training, including bedside instruction on how to integrate ultrasound findings into clinical care. Our findings reflect that study physicians reported a high level of confidence in their CPUS ultrasound exams after the training, and used their CPUS exams to influence their clinical impression and management plans in a significant number of cases.

The incremental diagnostic impact of portable ultrasound has been realized for over a decade, with many, including the World Health Organization, advocating for its use in resource-limited settings [2, 36]. The concept of a hand-held ultrasound or “sonoscope” used routinely in clinical practice has recently gained support, and with limited access to other diagnostic tests, the value of ultrasound in this population is magnified [1, 37–39]. For example, though ADHF is recognized as a common cause of dyspnea with high morbidity and mortality, there are significant limitations to the physical exam in the detection of ADHF as a cause for dyspnea, and brain natriuretic peptide (BNP) and chest roentgenogram are often not available and may ultimately delay diagnosis in limited-resource settings. Previous studies have described the value of focused cardiac ultrasound, lung ultrasound, or a combined protocol in resource-rich medical environments, including evidence-based support in international consensus conferences related to both clinicians performed focused echocardiography and point-of-care lung ultrasound [25, 40, 41].

Ultrasound-enhanced diagnosis of ADHF using the conventional echocardiography performed by a cardiologist or sonographer is the mainstay of current standard of care for diagnosis, and focused cardiac ultrasound is fast becoming a useful screening tool for non-cardiologists to initiate appropriate treatment and guide referrals in emergency departments in the US. The criterion standard for establishing the presence of left ventricular dysfunction for this study was the visual estimate of left ventricular systolic function using the images provided by the study physicians, a method that has been studied previously by Moore et al. to demonstrate emergency physician competence in bedside echocardiography for detection of heart failure in hypotensive patients and is supported as an established standard for the determination of ejection fraction within the cardiology literature [28, 42, 43].

Our study physicians had a high sensitivity and specificity for the detection of systolic left ventricular dysfunction in dyspneic patients compared with expert image review. Our test characteristics for accuracy and inter-rater reliability are similar to the high level of agreement seen between EM physicians and cardiologists or between cardiologists reviewing the same images [42, 43]. In addition, expert review of a single PLAX view produced in our study shows a PPV of 95 % and NPV of 93 % which would support its use as a particularly valuable screening test in patients with acute dyspnea.

The severity and prevalence of heart disease observed in our population of dyspneic patient deserve some attention. In our study, LV systolic dysfunction was found in 39.3 %, which is similar to that found in the US-based emergency populations, for example, from the “Breathing Not Properly” study published in the New England Journal of Medicine in 2002, investigators determined that of the 1500 patients studied, 47 % of them were diagnosed with CHF, and of those 2/3rd were diagnosed with ejection fraction <45 % indicating systolic dysfunction [33]. The average EF of our study patients with LVEF <50 % was 13.6 %, suggesting not only a high prevalence of previously undiagnosed left ventricular systolic dysfunction, but also increased severity upon presentation. The marked severity of LV dysfunction may relate to the barriers in access to medical care and low health literacy in this setting which likely delays presentation until the disease process is advanced.

Our study shows physicians readily integrated their CPUS findings, translating to a high proportion of patients whose clinical management and initial treating clinician impression were altered based on the CPUS. We found that the pre-CPUS treating physician’s clinical impression was changed in 15.3 % of patients, and 19.6 % of patients had an alteration in the management plan. This suggests that adoption of routine CPUS after a brief training intervention may be easy to achieve and may translate into immediate clinical impact. While focused cardiac ultrasound showing left ventricular systolic dysfunction is not equivalent to a diagnosis of the complex clinical syndrome of ADHF, the treating physicians used all available data, including history, exam findings, and other diagnostic testing to make their clinical diagnosis of heart failure [44].

Our study uniquely features a study population, including both adults and children, with pediatric patients representing 24.8 % (29/117) of our total population and 22/29 of these children were under the age of 5 years. While our CPUS protocol was the same for adults and children, we note that overall mortality rate between adults (13.7 %) and children (0 %) was different, and we found a lower clinical impact, including pediatric change in clinical impression (13.7 %) and change in management (3.4 %). While the most common discharge diagnosis for our study subjects with dyspnea in adults was ADHF (*N* = 40/88), for pediatric patients, pneumonia was the top cause (*N* = 16/29). Only two of our pediatric patients were found to have left ventricular systolic dysfunction, and both of these patients had very low ejection fractions of 10 %. Focal B-lines were seen in 5/29 children presenting with dyspnea, who went on to have a discharge diagnosis of pneumonia. Given these findings and the high prevalence of pneumonia diagnoses in our study, future research on CPUS protocols, including specific training on pneumonia, may be warranted.

## Limitations

Enrollment of patients was based on the availability of the treating physician and research assistant. Patients presenting with dyspnea in the evening hours or weekends were either not included or were enrolled the following day; Therefore, the interim management may have affected the ultrasound findings. Specifically, while we believe it is unlikely that significant improvement or change would occur to affect overall LV systolic function in patients with newly diagnosed ADHF given how low the average EF of the ADHF patients identified was (14 % average), it is possible that if properly diuresed overnight, B-lines as a sign of excess lung water would not be present on evaluation within hours of initial treatment. In our study, a clinical diagnosis, such as HF, was assigned by the treating clinician both prior to and after performing a limited cardiopulmonary ultrasound protocol. These diagnoses were made by the treating clinician in real time with the available information, and were not adjudicated by expert reviewers. Therefore, it remains possible that diagnostic inaccuracies would alter the sensitivity and specificity of the ultrasound findings for any of the specific diagnoses.

Our study included data on patients with severe mitral stenosis based on visual assessment of classic features of the mitral valve; however, as we did not include the use of continuous wave Doppler in the CPUS protocol, it is possible, we were not able to diagnose mild mitral stenosis without classic features.

We enrolled a mixed cohort of adults and children in our study to maximize information available, given that this is the first study of a unique CPUS protocol in dyspneic patients conducted in a limited-resource setting. Clinical assessment of dyspnea is largely similar in both adults and pediatric patients; however, specific data regarding pediatric findings of accessory muscle use and retractions were not recorded in our study, though all routine physical exam components would be captured in the physician overall impression of diagnosis pre-CPUS for adults and pediatric patients alike. Sonographic evaluation of pneumonia, a leading killer in both adults and children, was not included in our CPUS protocol, and may be a limitation to the interpretation of pulmonary portions of the ultrasound (B-lines).

We acknowledge that our CPUS protocol included only recording of lack of lung sliding as a specific and sensitive sign for pneumothorax, and did not include lack of comet-tail artifact, though this was included in the general ultrasound training received by study clinicians. This may have limited our ability to detect pneumothorax in this study.

Patients were enrolled in a single hospital in Haiti, which may limit the external validity and generalizability to other limited-resource settings.

## Conclusions

To our knowledge, this is the first prospective study of a combined cardiopulmonary ultrasound protocol in the diagnostic evaluation of patients with dyspnea in an international, limited-resource setting. Our study suggests that focused CPUS performed by general practitioners following a relatively brief training course can be performed with a high degree of accuracy and impact on the treating clinician’s impression and management in a significant proportion of patients. Severe underlying structural heart disease was common in this patient population, and a single parasternal long axis image had an excellent negative predictive value in excluding significant structural heart disease. Whenever possible, portable ultrasound should be used in evaluation of the dyspneic patient in resource-limited settings.
